# Review and Implications of Intergenerational Communication and Social Support in Chronic Disease Care and Participation in Health Research of Low-Income, Minority Older Adults in the United States

**DOI:** 10.3389/fpubh.2021.769731

**Published:** 2021-12-15

**Authors:** Joan A. Vaccaro, Trudy R. Gaillard, Ramces L. Marsilli

**Affiliations:** ^1^Department of Dietetics and Nutrition, Robert Stempel College of Public Health and Social Work, Florida International University, Miami, FL, United States; ^2^Nichole Wertheim College of Nursing and Health Sciences, Florida International University, Miami, FL, United States; ^3^Information and Research Service, Library Operations, Florida International University, Miami, FL, United States

**Keywords:** intergenerational communication, social support, health decisions, health research, older African Americans, Hispanics/Latino, chronic disease, geriatric care

## Abstract

**Background:** Health disparities disproportionally affect Black and Hispanic older US adults. Health research is needed to understand and eliminate these disparities; however, older adults, and particularly Black and Hispanic/Latino older adults are underrepresented in health research. Adult children have influenced health behavior and health outcomes of their older parents in several demographics in the US. Analysis of these studies can lead to a model for the development of interventions aimed at improving health and healthcare participation of older Black and Hispanic US adults.

**Objectives:** To review the role of intergenerational communication and social support in health behavior, health research, and health outcomes for older adults and to apply these findings toward a model for health interventions for Black and Hispanic US older adults.

**Methods:** An analytical narrative review and application toward an intervention model.

**Results:** Key topic areas were reviewed and analyzed by examining studies that applied forms of intergenerational communication and/or intergenerational social support with the goal of either improving health, disease management and/or participation in health research in populations world-wide. Next, a model for providing health interventions in older Black and Hispanic US adults was developed using strategies gleaned from the findings.

**Conclusion:** A model for health intervention for Black and Hispanic/Latino US older adults was presented based on an analytical review and intergenerational communication and/or social support. Qualitative data are necessary to understand the enablers and barriers of intergenerational communication and social support to improve health outcomes in these populations.

## Introduction

Low-income minority populations, in particular older adults, are underrepresented in health research and have poor health outcomes according to the National Institute of Aging (NIA) (1). Increasing recruitment of older, minority adults in health research is an ongoing strategy of the NIA to reduce health disparities (1). Intergenerational communication and/or intergenerational social support has the potential to influence health behavior and health outcomes for older adults. Research on intergenerational communication of families and other social support from minority populations could uncover the enablers and barriers, and motivators for health behavior and participation in health research; however, studies are lacking for intergenerational influence (influencers) on health behaviors of older minority populations in the US. Furthermore, there are few studies aimed at improving adherence to chronic disease care or encouraging participating in health research for older adults that apply the strategy of intergenerational communication and/or intergenerational social support in the US.

## Methods

This study was an analytical narrative review examining studies that applied forms of intergenerational communication and/or intergenerational social support with the goal of either improving health, disease management, or participation in health research in older populations world-wide. Mendeley library, Elsevier publisher was used to import research papers, reviews, and dissertations not restricting dates. Mendeley reference manager imports papers from multiple resources. Additional searches were performed using the Google search engine for reference articles provided by reviews and with the same key terms entered in Mendeley. Key terms included various combinations of the following: *intergenerational communication and/or intergenerational social support/ and or family intergenerational communication, and/or intergenerational relationships with, health, healthcare, adult child and elderly parent, recruitment of minorities in health research, disease management, hypertension, diabetes, cancer, diet, nutrition, physical activity, exercise, Hispanic/Latino, African American/Black*. Articles were selected based on relevance to the following components: (1) defining intergenerational communication and social support with respect to an adult child and their older parent; (2) intergenerational communication/social support on psychological health and self-rated health of older adults; (3) recruitment of minorities in health research; (4) intergenerational communication/social support in disease management, diet, and physical activity of older adults. Intergenerational communication/social support *w*ere defined, and a model was proposed incorporating cultural sensitivity and cultural humility strategies to improve intergenerational communication in US minority populations with the objective of improving the health of older adults.

## Results

The results are summarized below as separate topics.

### Origins and Trends of Intergenerational Communication and Family Social Support on Older Adults' Health

The degree of effectiveness of intergenerational communication on health and health behaviors of older adults is a subset of Family Communication Patterns Theory (FCPT). The premise of FCPT is that families share a social reality which influences their decisions, activities and interactions with others (2, 3). Thus, family communication is based upon the relationships and interactions of members within the family and the hierarchical relationship the family members may hold. Other theories have been used to study intergenerational communication such as social learning theory; however, the directionality observed has been from parent to child or grandparent to grandchild. This review investigated social support aspects of intergenerational communication on older adults' health and health behaviors with the caveat that relationships are often reciprocal.

Goals of intergenerational communication research proposed by O'Connor et al. (4) based on an Australia population were to: identify the enables of health common to both generations and those unique to each generation within a particular community; and, to develop, monitor, and evaluate specific approaches of intergenerational communication and available resources for effectivity of health improvement. In this study, community art therapy was used to bring younger and older adults to discuss health and well-being and how they can be brought together to enhance community strength. This study highlighted the respect and trust that are shared among generations and how this can be leveraged to strengthened community wellness.

In other studies, interrelationships among intergenerational communication, social support, and health of older adults have been studied primarily in Asian and Australian populations and sporadically in US populations. Key historical and geographical trends are represented in this paragraph. Intergenerational communication and social support of older adults and their adult children has been characterized as reciprocal in US populations in the 1980s (5). The patterns of communication and instrumental and emotional social support differ by living arrangement and physical proximity, frequency and quality of exchange, assistance, and support, and its contribution to psychological well-being. This study reported that well-educated, healthy, resourceful older adults were comfortable communicating with their adult children about their health and healthcare in the future (5).

The impact of modernization on intergenerational communication is complex yielding positive and negative consequences. Intergenerational communication of older adults from Thailand was shown to be augmented by technology despite the urbanization of their adult children and grandchildren (6). The authors reported use of the internet by older adults resulted in their effective communication contribution to their family which increased their sense of belonging, empowerment, and well-being (6). A US study suggested that moderated levels of support were psychologically beneficial and more accepted by widowed and unmarried parents compared to their counterparts; however too much support was counterproductive (7). In a rural community in the US, older adults reported better communication satisfaction and resources as compared to the younger generation. This study was self-reported perceptions and gives insight into how family members perceived communication not how they actually communicate (8).

There are several studies that reported the communication and interaction between grandparent and their grandchildren in the US. Grandparents were found to influence the pattern of communication of their children and grandchildren in the southwestern, rural region of the United States (9). Zhang and Hummert (10) reported that Western younger adults' stereotype of older adults as weak, sick, dependent, and not worthy of respect and Eastern younger adults not seeing the older adult as caring, loving, and of a higher status of them resulted in both cultures patronizing their parents. The authors further suggested that Eastern and Western adult children's patronizing is a danger to the psychological and physical health of older adults (10).

### Intergenerational Social Support on Psychological Health and Self-Rated Health of Older Adults

Intergenerational communication has been widely studied as a broad field; however, little is known about family intergenerational communication and its effect on health behavior and health outcomes in minority populations in the United States, such as Blacks and Hispanics. Family intergenerational studies of older adult's health have been studied primarily in Chinese and Australian populations with few on Chinese Americans, refugees and immigrant populations. Several studies demonstrated the psychological benefits for older adults who live in multigenerational households in China (11, 12), Thailand (6), and in California (13).

However, Silverstein and Bengtson (13) indicated that excess social support erodes confidence and causes psychological distress in a US population, which suggests that excessive support erodes independence and contributes to distress. Emotional support tends to reverse direction as parents age so that children are providing their older parents with intangible social support in a Northern European cohort (14). Intergenerational social support was positively associated with self-rated health for Chinese older adults (15) and Chinese American immigrants (16). A distinction should be considered between over-dependence on adult children's health advice and reciprocal and balanced relationships within the family when assessing the well-being, mental health, and community relationships of older adults (17).

### Recruitment of Minorities in Health Research

Disadvantaged groups, such as US racial/ethnic minorities, need to be included in health research in order to understand the mechanisms behind health disparities (18). There is a plethora of literature on the challenges of recruitment and retention of ethnic-racial minorities into health research. Immigrants and African Americans have heard of or had negative experiences with the healthcare system, and some have shown a distrust of healthcare professionals (19, 20). African American males, across all age groups, reported a lack of trust as a primary reason for their unwillingness to participate in health research (21). Approximately half of a sample of African American women stated that research in the United States is unethical (22). African American cancer patients were more likely to decline participation in a cancer clinical trial as compared to their White counterparts with family pressure and feelings of being overwhelmed as reasons for declining even after adjusting for phase in clinical trial, type of cancer, sex, and insurance (23). Researchers targeting this population need to understand the concerns and issues of this population for successful recruitment and retention.

### Intergeneration Communication and Social Support in Disease Management and Health Behavior

Communication is a dynamic and interactive process. There is focus in the literature on older adults as role models to encourage healthy eating for disease prevention for their adult children and grandchildren either with limited success (24) or a promise of a future strategy as with hypertension prevention in African American households (25, 26) but who do they consult for preventive health guidance? A number of studies focused on the effect of intergenerational communication and social support on health of older Chinese adults (27–29). Older Chinese adults were the respected elders to whom younger adult consulted for health guidance. Due to vastly different social systems and philosophies on elders, Asian intergenerational communication may have limited relevance to US cultures as half of US adult children live 10 or more miles from their older community-dwelling parents (30). Due to either living alone or having spouses with functional limitations, older, community-dwelling adults could prosper by social support of their adult children despite geographic barriers (30).

Intergenerational communication can be applied to enhance disease management in older adults through web-assisted technology. Younger adults (part of the family and/or community network) worked with community-dwelling older adults to develop a website to help African American older adults manage their diabetes (31). The barriers that emerged were technological skill and literacy. Enablers included having patience with older African Americans and small groups when teaching with web-based strategy to improve disease management (31).

### Cornerstone of Disease Management: Diet and Exercise and Intergenerational Communication

Adult children have been found to encourage their parents to adopt health behaviors and lifestyles in an effort to manage their chronic disease for Hispanic and African American older adults (32, 33). Intergenerational communication about healthy eating varied by race/ethnicity in terms of health encouragement for disease management for Italian, Anglo, and Asian families in Australia regardless of chronic disease history (34). Intergenerational exergaming improved intergenerational communication and motivation to exercise for older Chinese adults (35). Exergaming has been shown to be a viable motivator for exercise in older adults, while improving quality of life, empowerment, and intergenerational communication across populations (36); albeit, the role of intergenerational social support has yet to be investigated for underserved, low-income minority older adults as low socioeconomic status could be a barrier to access.

### Cultural Sensitivity, Cultural Humility, and the Development of Strategies for Health Interventions in Black and Hispanic Older Adults

Both cultural sensitivity and cultural humility are part of overall cultural competency in the delivery of health interventions. Culturally sensitive in health care is defined by the Office of Minority Health as upholding the main standard of being “appropriately responsive to the attitudes, feelings, or circumstances of groups of people that share a common and distinctive racial, national, religious, linguistic, or cultural heritage” (37).

Cultural humility is an attitude that reflects the healthcare professional's ongoing process of critically incorporating life-long learning, self-reflection (38). As such, clinicians practice cultural humility by having an ongoing internal dialog whereby their collaboration and negotiation with each client is driven by listening, learning, and negotiating (38). Culturally sensitivity and cultural humility can be integrated into interventions targeting intergenerational communication and social support to enhance healthy aging of older Hispanic and African Americans. This is particularly important as the US population of older adults are expected to outnumber children by the year 2030 (39).

## Discussion

There were few studies that examined how older Hispanic and/or Black adults use advice from adult children for their health behavior, health outcomes, or participation in health research. Lack of trust of the healthcare system, fear of research being unethical, and feelings of being overwhelmed have prevented US minorities from participating in clinical trials. This review was timely and relevant since it investigated the role of intergenerational communication and social support in health behavior, health research, and health outcomes for older adults worldwide with an emphasis on Black and Hispanic populations was reviewed.

Intergenerational communication and social support were characterized as reciprocal in the US in the 1980's (5); however, this may have changed over 40 years. Older adults may not feel comfortable receiving guidance from their adult children since Zhang and Hummert (10) reported that many young US adults characterize older adults as weak, sick, and dependent and not worthy of respect. Moreover, young adults who have a negative stereotype about older adults could respond by providing excess social support. Psychological distress and eroded confidence were the outcomes of excess social support given to US older adults (11). Web-based intervention programs utilizing intergenerational communication in Western cultures have been shown to have some benefits to help older adults eat healthier (33), increase performance of safe exercise (33), and manage chronic disease (diabetes) (31) while uncovering barriers for participation. Of promise are several pinnacle studies where adult children helped their older parents manage their chronic diseases in Hispanic (32) and African American (31, 33) populations.

**Figure 1:**
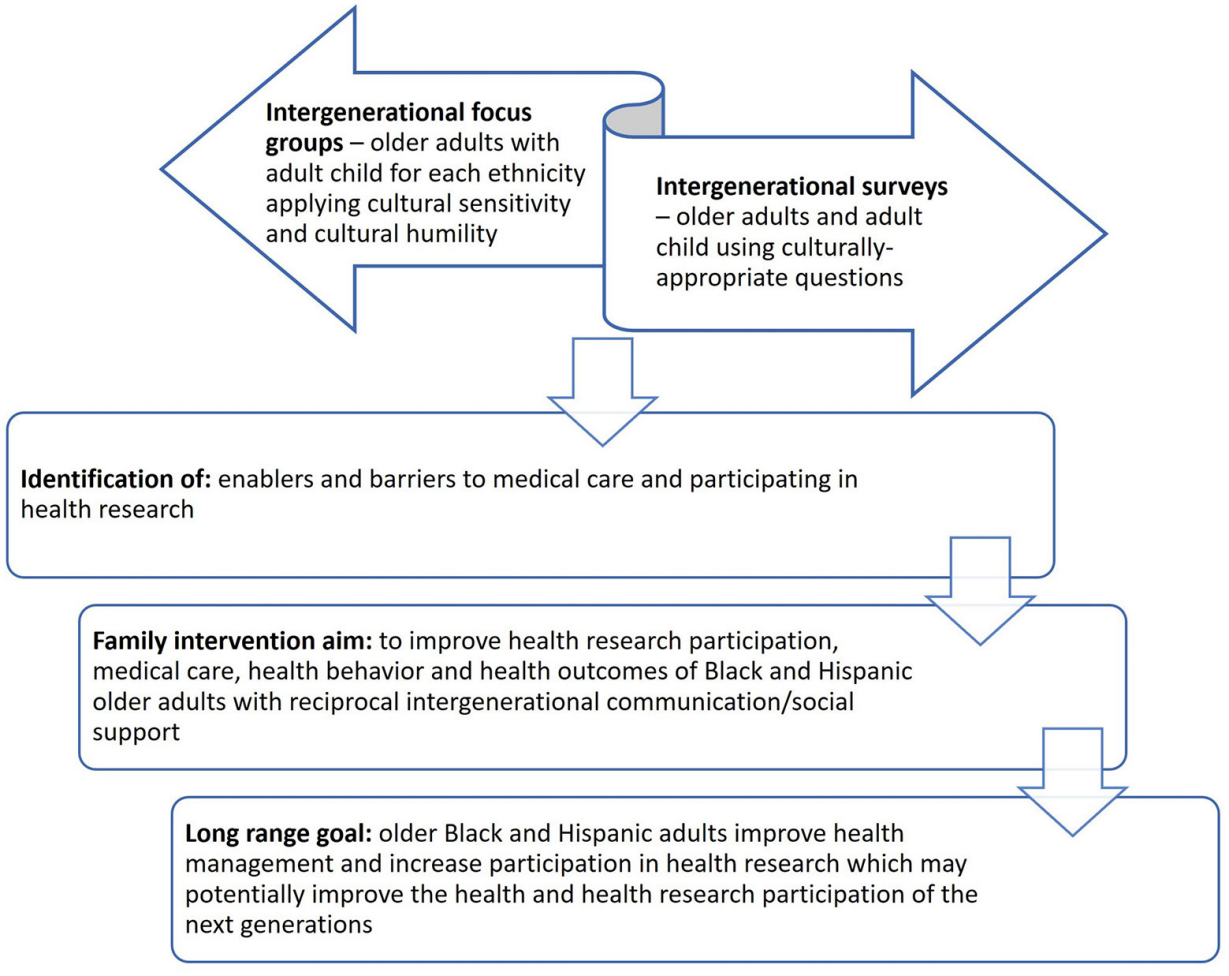
Strategy of intergenerational communication and social support for health improvements in Black and Hispanic older adults.

The findings of this review, together with cultural sensitivity and cultural humility were incorporated in the proposed model (Figure 1). The result is that strategies to improve family intergenerational communication of Black and Hispanic older adults require separate focus groups for each ethnicity within Black and Hispanic populations, to address their unique concerns. Induced enablers and barriers from qualitative research (focus groups) and resulting survey interpretation are proposed to improve medical care, health behaviors, and health outcomes. The overarching goal is to increase participation of minority older adults in healthcare and healthcare research and improve the health of the next generations.

This study had several limitations. First, subtle health behavior changes due to intergenerational communication and social support for older adults may have been missed due to focus of the subtopics. Second, the search terms used may not have exhausted all the possibilities of intergenerational communication and social support. Finally, the analysis is based on the education and experience of the authors in the field of health.

## Conclusion

An analytical review of the literature indicated promise in health participation and potential health improvements for older Black and Hispanic adults by seeking guidance from their adult children and grandchildren, particularly in areas where health is connected to the virtual world. A few promising studies of adult children encouraging their parents to adopt health behavior in an effort to manage their chronic diseases were found for Hispanic and African American older adults. This focused review builds upon the finding from other cultures due to a gap in the literature for US minorities. Qualitative research is necessary to direct future programs aimed at reducing health disparities due to the wide differences in intergenerational influences across the ethnic racial groups in the US. Effective intergenerational communication and social support could potential be beneficial in health improvement for older adults for each culture within Black (Caribbean-American, African American) and Hispanic (Cuban-American, Puerto Rican-American, Central-American, South-American) ethnicities. This is due to the wide differences in attitudes and behaviors across cultures.

## Author Contributions

TG and JV contributed equally to the literature search, literature analysis, data integration, and writing the manuscript. RM contributed to the literature search and critically reviewed the manuscript. All authors contributed to the article and approved the submitted version.

## Funding

Research reported in this publication was supported by the National Institute on Aging of the National Institutes of Health under Award Number #1R24AG067951-01.

## Author Disclaimer

The content is solely the responsibility of the authors and does not necessarily represent the official views of the National Institutes of Health.

## Conflict of Interest

The authors declare that the research was conducted in the absence of any commercial or financial relationships that could be construed as a potential conflict of interest.

## Publisher's Note

All claims expressed in this article are solely those of the authors and do not necessarily represent those of their affiliated organizations, or those of the publisher, the editors and the reviewers. Any product that may be evaluated in this article, or claim that may be made by its manufacturer, is not guaranteed or endorsed by the publisher.
